# Assessing Vitamins, Minerals and Supplements Marketed to Children in Canada

**DOI:** 10.3390/ijerph16224326

**Published:** 2019-11-06

**Authors:** Charlene Elliott

**Affiliations:** Department of Communication, Media, and Film, University of Calgary, Calgary, AB T2N 1N4, Canada; charlene.elliott@ucalgary.ca; Tel.: +1-403-220-3180

**Keywords:** vitamin, child, pediatric, marketing, nutrient, mineral, supplement

## Abstract

Given the growth of supplements specifically designed for children in Canada, this study examines the nutrient levels of these products, and evaluates them in light of the US Health and Medical Division (HMD)—formerly the Institute of Medicine—and Health Canada’s recommendations. Content analysis was used to document the nutrient levels of child-targeted vitamins, minerals and fish oils/omega-3s (n = 80) in Calgary, Alberta, Canada. Products were assessed according to HMD and Health Canada dosage recommendations for children, and the percentage of Estimate Average Requirements (EAR), Adequate Intakes (AI), and Tolerable Upper Intakes Level (UL) calculated. Median EAR/AI/UL percentages and quartiles were calculated for each nutrient, and estimates for the adequate intake recommendations plotted with box plots. Sixty five percent of the products assessed were multivitamins; the median dose was higher than AI recommendations for vitamins A, B6, B12, and C, as well as thiamin, riboflavin, pantothenic acid, and biotin. Substantial variation in vitamin, mineral, or fish oil dosage was found between similar supplements—with nutrients such as vitamin B12 ranging from 83% to 5557% of AI. Such findings matter because the very existence of these products suggests that children should be taking them, yet more research is needed on their potential (adverse) effects over both the short and long term. The substantial variation in dosages between products also raises questions about the (perhaps unnecessary) fortification of our children, as well as the expectations that parents know—or are even aware of—appropriate nutrient levels for their kids.

## 1. Introduction

Heightened consumer interest in health and wellness has prompted a significant growth in dietary supplements. Globally, the dietary supplements market is projected to reach USD 278.02 billion by 2024, with vitamins-based supplements comprising a substantial part of this revenue [1]. In 2018, the global vitamins market was valued at USD 5.18 billion [2], and numerous market-based research reports laud its size and future growth potential [1,2,3,4].

One expanding segment of this “vitamins market” is vitamins, minerals, and supplements targeted at children. Gummy and chewable vitamins packaged with child-friendly appeals, such as *Disney Multivitamin Gummies Disney Princesses* and *Star Wars Multivitamin Gummies* are now a commonplace, as are supplements like *Kids Only! Omega-3* in “fruit blast” flavors, or ones promoted as “Fun for kids!” or with “Fun superhero shapes!” Such packaging suggests that children should receive dietary supplements “specially designed for them”—even though professional medical organizations do not recommend vitamin use in healthy children [5].

Despite the growing number of child-targeted vitamins, minerals, and supplements, few studies examine their risks [6,7,8,9,10,11,12,13,14] and only one published study examines the labeled vitamin content of dietary supplements for children. This American study [15] analyses vitamin dosages for children under age 4, but does not examine the supplements specifically marketed as for children or address minerals or fish oils. Given this research gap, the purpose of our study is to examine the nutrient levels of vitamins, minerals, and other supplements specifically designed for children in the Canadian marketplace, and to evaluate them in light of the US Health and Medicine Division (HMD) (formerly the Institute of Medicine) and Health Canada’s recommendations. (Note: as per HMD stipulations, reports issued prior to June 30, 2015 are to be cited as IOM reports in perpetuity. As such, IOM is used throughout this article.).

## 2. Methods

Content analysis was used to create a profile of the vitamins, minerals, and supplements designed for children in the Canadian marketplace. Pediatric vitamins and supplements were purchased for coding in the Spring of 2017; 58 variables were recorded for each product by the lead researcher and a trained graduate student. Upon completion, one graduate student and one postdoctoral scholar independently reviewed the coding for accuracy. Each case was identified in terms of brand, product name, type of supplement, recommended dosage, and specific vitamin/mineral/fish oil dosage per serving. Variables related to package semiotics, verbal claims, and supplement shape, form (gummy/chewable), and colour were also recorded. For the purposes of this article, however, we focus specifically on the dosage ranges in these products.

Supplements were included in the study if the product packaging: (a) claimed it was for children or “kids” (including products with “kid” in the brand or product name, such as *IronKids Essential* or *SmartyPants Kids Complete*), or (b) featured child-oriented cartoons or tie-ins with children’s toys or media culture, including television shows and movies. To ensure the dataset was comprehensive when it comes to brick and mortar stores, all relevant products were purchased from eight venues (four major supermarket chains, two drug stores, one department store, and a warehouse retailer). Specific venues included: Safeway, Co-op, The Real Canadian Superstore, Sobeys, London Drugs, Shoppers Drug Mart, and Wal-Mart. The selected venues represented Canada’s three largest retailers: Loblaws Companies Ltd., Costco and Sobeys Inc [16] (Note that Loblaws Companies owns the Real Canadian Superstore and Shoppers Drug Mart. Sobeys owns Safeway.). Wal-Mart Canada was included since it is another top retailer in the country, while London Drugs is a Canadian-owned retail store chain with a strong presence in Western Canada. Stores were visited on multiple occasions to ensure all relevant products were collected. Duplicate products, as well as products in different size formats, liquids/tinctures, and infant vitamins/supplements were not included.

Products were assessed in light of the recommended dosage for two intended user groups: children aged 2–3, and children aged 4 and older (as these were the age ranges most frequently found on the label). Each dietary supplement label was separated by its contents into a database (SPSS v24, IBM Corp, Armonk, NY, USA). Expected vitamin intake was calculated by examining each product and multiplying the dosage of each gummy or tablet by the number of consuming times recommended per day on the label, for each specific age group.

Vitamin/mineral intake amounts were then evaluated in light of the IOM’s Dietary Reference Intakes (DRIs) tables [16], which “provide the scientific basis for the development of food guidelines in both the United States and Canada” [17], and specify nutrient reference values on the basis of age and gender for over 40 nutrient substances. For Vitamin D and calcium, Health Canada’s updated Dietary Reference Intakes [18] were used. See Table 1 and Table 2 for a breakdown of these dosage recommendations.

Specifically, nutrient intake amounts were divided by the IOM and Health Canada daily Estimated Average Requirements (EAR, defined as the average daily nutrient intake level estimated to meet the nutrient needs of half of healthy individuals in a group), Adequate Intakes (AI, defined as the estimated nutritional intake suitable to maintain adequate nutritional state), and Tolerable Upper Level of Intake (UL, highest average intake that is likely to pose no risk) to reveal the percentage of recommended EAR/AI/UL. The median EAR/AI/UL percentages and quartiles were calculated for each individual nutrient as averages are more susceptible to statistical outliers. Estimates for the adequate intake recommendations were then plotted with boxplots. For fish oil products containing docosahexaenoic acid (DHA) and eicosapentaenoic acid (EPA), Health Canada’s monograph on Fish Oil recommends a combined daily dose range of 100–1500 mg/day for children one to eight years old [19]. Given that there is no current recommended AI dosage provided by the IOM or Health Canada we instead analysed the dosage *range* suggested in each of these products, to reveal variances in recommended consumption levels by brand (i.e., this analysis calculates the median and quartile estimates for the recommended dose of each product instead of the percent of the recommended threshold). Statistical analyses were conducted using R Statistical and Analysis Software, (R Foundation for Statistical Computing, Vienna, Austria) [20].

## 3. Results

Eighty products were purchased for analysis. Fifty-five products (69%) listed a recommended dose specifically for children ages 2–3, 77 products (96%) provided recommended doses for children ages 4 and up, and three products (4%) listed 6 years as the youngest age for consumption. The *number* of gummies/tablets recommended for a child’s daily consumption varied substantially, from one to 15 per day. A majority of the products (65%) were multivitamins; the rest were single vitamins, vitamin/mineral combinations, or products labelled “immune support” or “greens”. See Table 3 for a breakdown of the products by supplement type.

**Figure 1:**
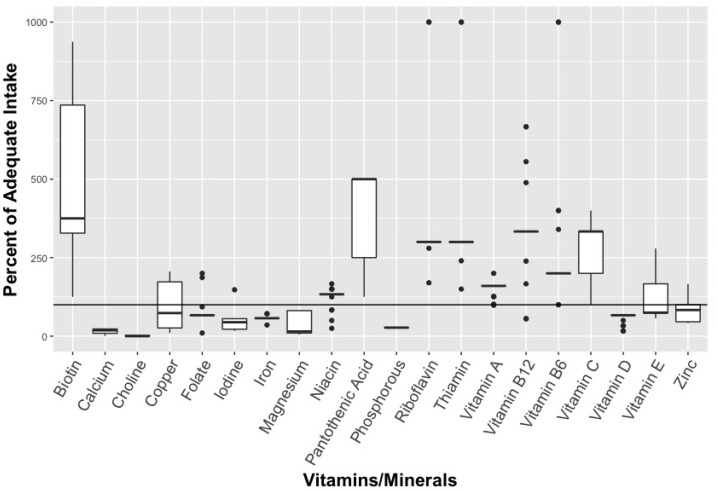
Percent of recommended vitamins/minerals for 2–3 year old in multivitamin supplements (n = 32). * Solid line represents median estimate, with top and bottom of box representing quartiles (if sufficient data). Y—axis was truncated at 1000% to allow appropriate visualization of the estimates.

**Figure 2:**
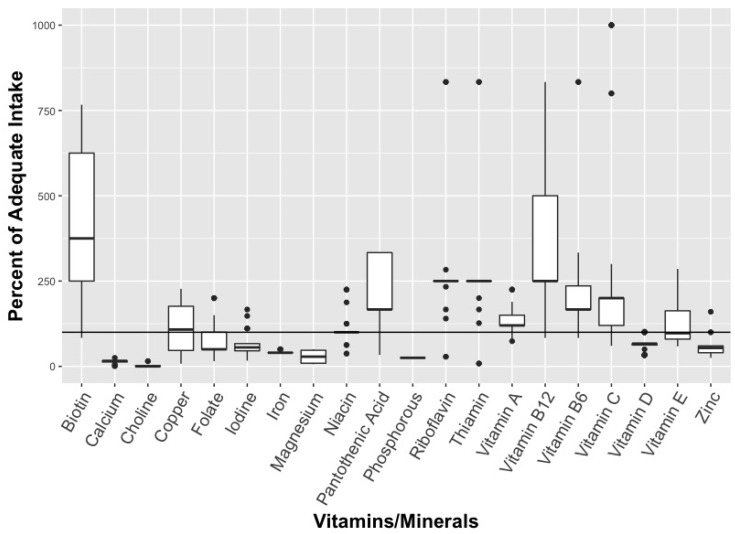
Percent of recommended vitamins/minerals for children aged 4 and up in multivitamin supplements (n = 52). * Solid line represents median estimate, with top and bottom of box representing quartiles (if sufficient data). Y—axis was truncated at 1000% to allow appropriate visualization of the estimates.

When it comes to multivitamins, the median dose for many nutrients were higher than the IOM recommendations for adequate intake (AI) (see Figure 1 and Figure 2).

None of the products analysed exceeded the Tolerable Upper Intakes Levels (UL) for children. For 2–3 year olds, the median dose for biotin, pantothenic acid, riboflavin, and thiamin is 375%, 500%, 300% and 300% of AI respectively for multivitamins, while the median dose for Vitamins A, B6, B12, and C also exceeded the AI dose—ranging from 160% AI (Vitamin A) to 333% (Vitamins B12 and C) (Figure 1). Similarly, for children 4 and older, the median dose for biotin, pantothenic acid, riboflavin, and thiamin were 375%, 167%, 250% and 250% of AI respectively, while the median dose for Vitamins A, B6, B12, and C was higher than the AI dose (Figure 2). Calcium, choline, iron, and phosphorus had notably low median doses for both children aged 2–3 and children 4 years and older (Figure 1 and Figure 2). For iron, the median dose was 57% of AI for children aged 2–3 and 40% in children 4 years or older, or 10% of UL in both age groups. When it comes to EAR, the median dose was in excess for Vitamins A, B6, B12, and C, niacin, riboflavin, thiamin, and iron.

These multivitamins had a considerable range of nutrients, depending on the product. Dose ranges for Vitamin B6, B12, and C, for example, extended from less than or equal to the AI to over 1000% of the AI for both children aged 2–3 and 4 years or older (Figure 1 and Figure 2). In contrast, minerals tended to have less variation in dose between products, with the exception of biotin which ranged from 125%–938% of AI for children aged 2–3 and from 83%–1250% for children 4 years and older. The maximum dose for any nutrient in children aged 2–3 was Vitamin C at 1923% of AI and 1667% of recommended AI for ages 4 and older (Figure 1). The maximum dose for children 4 years and older was for Vitamin B12 at 6668% of EAR and 5557% of recommended AI (see Figure 2).

**Figure 3:**
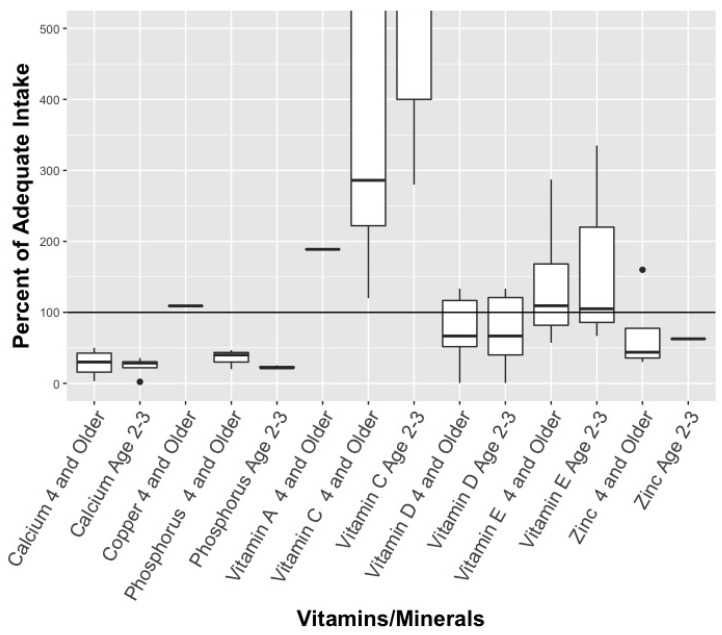
Percent of recommended vitamins/minerals for 2–3 year old and children ages 4+ in non-multivitamin supplements. * Solid line represents median estimate, with top and bottom of box representing quartiles (if sufficient data). Y—axis was truncated at 1000% to allow appropriate visualization of the estimates.

For the non-multivitamins analysed, no nutrients exceeded the UL for vitamins or minerals, and the median dose was less than the AI for most nutrients (see Figure 3). However, Vitamin C exceeded the AI with a median dose of 1667% for 2–3 year old and a median dose of 286% for children 4 years and older.

The fish oil, DHA, and EPA supplements matched the recommended amounts for children ages 2–3, while exceeding the recommendations for children 4 years and older (Figure 3). Recommended fish oil dosages (from individual products) for ages 4 and up ranged substantially, from 340 mg to 2250 mg per day. Similarly, recommended dosages of DHA ranged from 44 mg to 450 mg, depending on the product, while EPA dosages ranged from 20 mg to 900 mg. Worth noting is that the highest number of ‘gummies’ recommended for children’s daily consumption was in this category: a jar of Omega-3 gummies recommended that children 3 years and older should “chew 15 gummies daily” for the “**Development of brain, eyes and nerves**” (bolding on label).

## 4. Discussion

In 2017, global market research firm Innova penned an article on children and dietary supplements with the headline “Little Kids, Big Market” [21]. Innova noted the “growing market” of supplements and parents’ interest in using them to improve their children’s general health, to close “potential dietary/nutritional gaps” and to boost “cognitive development” [21]. Such parental interest in nutritional supplementation certainly seems to be the case: over 33% of American children take vitamins [22,23] and 45% of Canadian children (aged 1–8) are given nutritional supplements [24]. That over 1/3 American children and close to half of Canadian children take supplements is noteworthy given that the American Academy of Pediatrics does not endorse vitamin use in healthy children [5].

Even so, our study reveals that the Canadian marketplace offers myriad supplements designed for children. None of the 80 products analyzed exceed the UL for children. However, like previous studies [15], we found that the pediatric multivitamins analyzed contained dosages significantly above the AI—in our case, the median dose of products was as high as 500%. This finding is particularly noteworthy because, unlike previous studies [15], calculating the median EAR/AI/UL percentages and quartiles for each nutrient makes the findings less likely to be influenced by statistical outliers. Considering the median dose, as well as the outliers, is important for several reasons. First, there is the simple question of excessive nutrient exposure writ large. Our findings reveal that the median dose levels for biotin, pantothenic acid, riboflavin, thiamin, Vitamin A, Vitamin B6, Vitamin B12, and C far exceed the AI—and the dosage levels recommended on the product labels appear inattentive to this fact. Previous studies have pointed to the potential for excessive intake of Vitamin A, folate, and zinc for very young children with multivitamin consumption [6], also cautioning about the potential for overconsumption of Vitamin D or A due to product packaging/instructions or because of product appeal (i.e., candy-like form) [8,9,10]. Other studies have tracked the proportion of Emergency Department admissions for supplement use, revealing that intentional (i.e., not unsupervised ingestion) consumption of vitamins and mineral products were associated with 67% of adverse events in American children [12], and that possible natural health products interactions (with each other or with conventional medicines) linked to 16% of children’s emergency visits tracked in a Canadian hospital [13]. As a whole, this literature base points to the possible “needless supplementation” of children and the critical issue of child vitamin exposure [7].

Secondly, the outliers in our study underscore the careful attention demanded of parents when it comes to selecting a supplement. *Jamieson Multi Vitamin and Mineral Supplement for Kids Gummies*, for example, offers a dose of 0.5 mg/day of vitamin B6 for children age 4+, while *Natural Source Kids Chewable Fruit & Veggie Enriched Multi (NSKidsChewable)* and *Treehouse “Max & Ruby” Chewable Multivitamin with Minerals (TMRC)* contain a dose of 5 mg/day. Parents will give their child ten times the vitamin B6—1000% more—should they purchase the latter multivitamins over the former. Similarly, *Nickelodeon Multivitamins (Dora & Friends)*, *Ironkids Essentials Gummies Multivitamins* and *Nickelodeon Multivitamin Gummies (SpongeBob SquarePants)* all contain a vitamin B12 dose of 1 mcg/day for children age 4+, while *NSKidsChewable* and *TMRC* contains a dose of 10 mcg/day. In this case, parents will give their children ten-times the vitamin B12 (1111% of the recommended AI) if they purchase *NSKidsChewable*. When it comes to biotin, the lowest daily dosage for 2–3 years old is 10 mcg with two products and the highest is 75 mcg with three similarly packaged products. In a final example, calcium dosages for children (ages 4+) ranged from 10 mg to 500 mg.

Such substantial variation in dosage depending on the supplement is problematic, given that all of the products use the same kinds of appeals on the package. There is no reason for a parent to assume that *Nickelodeon “Dora & Friends” Multivitamins* are any different from *Treehouse “Max & Ruby” Multivitamins*, for example. Both similarly target to children, come in gummy form, and depict characters from popular children’s cartoons. While some research exists on the overconsumption of candy-like chewable supplements by children—specifically the risk of overdose or toxicity in children because a child gained access to the chewable/gummy vitamins and ate them as if candy [8,25]—no studies focus on the critical point raised above; namely, the substantial range of vitamins in terms of dose that a child might get due to a parent purchasing one child-targeted multivitamin product over another. This point matters because there is little reason to expect parents to know the correct intake of various vitamins and minerals for their children, or to scrutinize competing multivitamins/multi-minerals for different individual nutrient levels. Consider, for example: *Treehouse Gummies Multivitamin with Minerals (Max & Ruby)* and *Treehouse Chewable Multivitamin with Minerals (Max & Ruby)*. Both packages are virtually identical, with blue packaging, a prominent cartoon image of the bunny Max on the font, and identifying fruit flavors. The only difference is one product is in gummy form, the other is in chewable form, and the latter packaging states “For Active and Growing Children”. Both products recommend taking two per day. By simply choosing chewable multivitamins over the gummy form, a parent would give their child very different dosages: 200 mg vs. 30 mg of vitamin C; 10 mg vs. 5.6 mg pantothenic acid; 10 mcg vs. 66 mcg biotin; and 130 mg vs. 10 mg calcium—just to name a few discrepancies. The chewable version contains higher levels of vitamins in most cases (but not all), and also contains iron.

Beyond this, the dosages are further complicated by the number of gummies or chewables required to reach a recommended dose. Consider *Disney’s Finding Nemo Omega-3 Gummies*: the recommended “serving” of four gummies results in roughly the same fish oil, DHA and EPA dosage as the 15 gummies recommended by *Max & Ruby Omega-3* gummies. A “normal” daily dosage thus ranges from four to 15, depending on the product. One might reasonably question the logic of feeding a child 15 gummy candies per day to “improve” his or her health—as well as how the different number of gummies to reach the same dose (depending on the product) is problematic from a public health perspective.

Finally, there is the broader issue of how the presence and sheer quantity of these products work to communicate to parents that supplementation is both normal and desirable. The very existence of *Disney “Mickey Mouse” Immune Support Gummies* works to suggest that parents should consider boosting their children’s immunity in this fashion. Moreover, the idea that children should have supplements “specially designed for them”—and ones signaled by direct claims to “fun” and licensed characters (such as The Avengers, Franklin, Barbie, etc.) simply extends other trends found in children’s food marketing [26,27,28]. Such normalization is problematic because supplements (with their concentrated doses of vitamins, minerals, and fish oils) should not be treated in the same fashion as food. As Ethan and colleagues observe in their study of product packing marketing strategies used to promote pediatric vitamins in the United States, “children are in need of greater protection from these products that are often candy-like in appearance and marketed as flavorful and ‘fun to eat’” [22].

To be clear, this research does not question the importance of getting adequate vitamins, minerals and omega-3s in the diet. When it comes to children, a growing evidence base documents that the health of many young people is compromised by what they are eating. A Canadian study reports that 55% of children’s total daily energy intake comes from ultra-processed food, which creates a challenge to getting adequate vitamins and minerals [29]. Yet the study authors recommend quality, unprocessed foods as the source of vitamins and minerals, not gummy vitamins [29]. Similarly, a position paper from the Academy of Nutrition and Dietetics on “Micronutrient Supplementation” does not make specific recommendations for children ages 2+ (with the exception of Vitamin D) [30], instead affirming that “children aged two to eight years who use dietary supplements are more likely than older children to meet the micronutrient recommendations from foods alone” [30]. Cautioning that use of dietary supplements “increases the prevalence of usual nutrient intakes above the UL for iron, zinc, copper, selenium, folate, and vitamins A and C”, the paper expresses particular concern about 2 to 8 year-old micronutrient supplement users, as they exceed the UL for zinc (84%), folic acid (71%), and vitamin A (72%) [30]. In brief, consumers need to consider nutrient intake from all foods and beverages, as well as supplements. Inadequate nutritional intake is a problem, but so is (as Tarasuk notes of nutrition-related marketing to consumers) “gratuitous fortification” [31].

### Strengths and Limitations

This study is the first to the examine the supplements specifically designed as for children available in the Canadian marketplace, and to evaluate them in light of current recommendations. Study strengths include analyzing a comprehensive dataset of child-targeted products, visiting the stores several times to ensure all relevant products were collected, and having multiple research assistants recheck the data. Another study strength is that the products were physically collected from brick and mortar stores, and, therefore, ones currently available for purchase by Canadians. Relying on websites as a source for data collection is problematic because products no longer available may still be listed online. By going into the stores, we capture the products that Canadian shoppers physically encounter. Finally, calculating the median EAR/AI/UL percentages and quartiles for each individual nutrient (rather than relying on averages) shields the findings from the impact of statistical outliers. While the final dataset is representative—products were collected from nine major retailers with a national presence, and the same national brands are sold across the country—an examination of the child-targeted products online, as well as those in health food stores, would provide interesting comparative data. Moreover, these findings speak to the Canadian marketplace; the nutrient levels and marketing strategies are not necessarily generalizable to low- and middle-income countries.

## 5. Conclusions

This study is the first of its kind to examine the nutrient levels of vitamins, minerals and dietary supplements specifically marketed as for children in the Canadian retail environment. On the one hand, the promotion of gummy and chewable supplements using cartoon and licensed characters and claims that the products are “fun to eat” simply extends contemporary strategies found in child-targeted packaged foods. Yet vitamins are not foods, nor are they regulated as foods in Canada, and this study questions the logic of recommending that children consume up to 15 gummy candies per day in order to “improve” their health.

Beyond this, the substantial variation in dosage depending on the supplement is problematic, given that all of these supplements use the same kinds of appeals on the package. Note that HMD (formerly IOM) does not set ULs for vitamin B12, vitamin K, thiamin, riboflavin, pantothenic acid, or biotin for children aged one to eight due to “lack of data of adverse effects in this age group and concern with regard to lack of ability to handle excess amounts.” It recommends that children rely on food only to prevent high intake [32]. Yet our study reveals that the median dose levels for all of these nutrients (with the exception of vitamin K) far exceed the AI recommendations, as did vitamin A, B6, and C. Moreover, the outliers reveal the extra expectations of caregivers to actually know the AI and to meticulously compare between supplements—expectations unlikely to be met. The expanding quantity of child-targeted dietary supplements works to communicate to parents that such supplementation is both normal and necessary, but such marketing remains at odds with current HMD and Health Canada recommendations. Suggestions for future research include focusing on the substantial variation in dosages between similar products, which would be particularly useful for policy (i.e., is there a need for greater standardization of these products) and useful for health professionals when it comes to making recommendations to parents. Qualitative research on parent perspectives on supplementation for children, and the products they use, would also be fruitful.

## Figures and Tables

**Table 1:** Vitamin specific dosage recommended by the Institute of Medicine and Health Canada for Estimated Average Requirements (EAR), Recommended Dietary Allowances and Adequate Intakes (AI), and Tolerable Upper Intakes Levels (UL).

	Vitamin A (mcg/day)	Vitamin B6 (mg/d)	Vitamin B12 (mcg/d)	Vitamin C (mg/d)	Vitamin D (mcg/d)	Vitamin E (mg/d)
Children ages 2–3						
EAR	210	0.4	0.7	13	10	5
AI	300	0.5	0.9	15	15	6
UL	600	30	NA	400	63	200
Children ages 4 and older						
EAR	210	0.5	0.9	22	10	6
AI	300	30	NA	25	15	7
UL	600			650	75	300

**Table 2:** Mineral-specific dosage recommended by the Institute of Medicine and Health Canada for Estimated Average Requirements (EAR), Recommended Dietary Allowances and Adequate Intakes (AI), and Tolerable Upper Intakes Levels (UL).

	Biotin (mcg/d)	Calcium (mg/d)	Choline (mg/d)	Copper (mcg/d)	Folate (mcg/d)	Iodine (mcg/d)	Iron (mg/d)	Magnesium (mg/d)	Niacin (mg/d)	Pantothenic Acid (mg/d)	Phosphorus (mg/d)	Thiamin (mg/d)	Riboflavin (mg/d)	Zinc (mg/d)
Children ages 2–3														
EAR	NA	500	NA	260	120	65	3	65	5	NA	380	0.4	0.4	2.5
AI	8	700	200	340	150	90	7	80	6	2	460	0.5	0.5	3
UL	NA	2500	1000	1000	300	200	40	65	10	NA	3000	NA	NA	7
Children ages 4 and older														
EAR	NA	800	NA	340	160	65	4.1	110	6	NA	405	0.5	0.5	4
AI	12	1000	250	440	200	90	10	130	8	3	500	0.6	0.6	5
UL	NA	2500	1000	3000	400	300	40	110	15	NA	3000	NA	NA	12

**Table 3:** Breakdown of Vitamin/mineral/supplement (n = 80).

Product Type	n	(%)
Multivitamin	52	65.0%
Vitamin C	3	3.8%
Calcium (including Calcium + D)	3	3.8%
Omega 3/DHA Omega 3/Fish Oil Supplement	11	13.8%
Vitamin D	6	7.5%
Immune Support	3	3.8%
Greens	1	1.3%
Zinc	1	1.3%
